# Association of the Asp312Asn and Lys751Gln polymorphisms in the *XPD* gene with the risk of non-Hodgkin’s lymphoma: evidence from a meta-analysis

**DOI:** 10.1186/s40880-015-0001-2

**Published:** 2015-03-05

**Authors:** Shen Chen, Jin-Hong Zhu, Fang Wang, Shao-Yi Huang, Wen-Qiong Xue, Zhuo Cui, Jing He, Wei-Hua Jia

**Affiliations:** State Key Laboratory of Oncology in South China, Collaborative Innovation Center for Cancer Medicine, Department of Experimental Research, Sun Yat-sen University Cancer Center, 651 Dongfeng Road East, Guangzhou, Guangdong 510060 P. R. China; Molecular Epidemiology Lab and Laboratory Medicine, Harbin Medical University Cancer Hospital, Harbin, Heilongjiang 150040 P. R. China; Department of Statistics, University of California, Berkeley, CA 94702 USA

**Keywords:** *XPD* gene, DNA repair, Polymorphism, Non-Hodgkin’s lymphoma, Meta-analysis

## Abstract

Polymorphisms in DNA repair genes may alter DNA repair capacity and, consequently, lead to genetic instability and carcinogenesis. Several studies have investigated the association of the Asp312Asn and Lys751Gln polymorphisms in the xeroderma pigmentosum complementation group D (*XPD*) gene with the risk of non-Hodgkin’s lymphoma (NHL), but the conclusions have been inconsistent. Therefore, we performed this meta-analysis to more precisely estimate these relationships. A systematic literature search was performed using the PubMed, Embase, and Chinese Biomedical (CBM) databases. Ultimately, 6 studies of Asp312Asn, comprising 3,095 cases and 3,306 controls, and 7 studies of Lys751Gln, consisting of 3,249 cases and 3,676 controls, were included. Pooled odds ratios (ORs) and 95% confidence intervals (CIs) were calculated to assess the strength of each association. Overall, no association was observed between the Asp312Asn polymorphism and NHL risk (homozygous: OR = 1.11, 95% CI = 0.94-1.32; heterozygous: OR = 1.00, 95% CI = 0.89-1.11; recessive: OR = 1.12, 95% CI = 0.95-1.31; dominant: OR = 1.02, 95% CI = 0.92-1.13; and allele comparison: OR = 1.04, 95% CI = 0.96-1.12) or between the Lys751Gln polymorphism and NHL risk (homozygous: OR = 0.97, 95% CI = 0.83-1.15; heterozygous: OR = 0.96, 95% CI = 0.86-1.06; recessive: OR = 1.00, 95% CI = 0.86-1.16; dominant: OR = 0.96, 95% CI = 0.87-1.06; and allele comparison: OR = 0.98, 95% CI = 0.91-1.05). Furthermore, subgroup analyses did not reveal any association between these polymorphisms and ethnicity, the source of the controls, or the NHL subtype. These results indicated that neither the Asp312Asn nor Lys751Gln *XPD* polymorphism was related to NHL risk. Large and well-designed prospective studies are required to confirm this finding.

## Background

Non-Hodgkin’s lymphoma (NHL) represents a large heterogeneous group of B-cell and T-cell lymphomas characterized by uncontrolled malignant clonal expansion. Approximately 80%-90% of all NHLs originate from B cells, i.e., B-cell lymphomas, which are further categorized into two major subtypes: diffuse large B-cell lymphoma (DLBCL) and follicular lymphoma (FL) [1]. The incidence of NHL continues to increase worldwide, and an estimated 355,900 new NHL cases and 191,400 NHL deaths occurred in 2008 [2]. Moreover, NHL imposes a heavy burden on patients by reducing quality of life and work ability and by increasing disability. The etiology of NHL has yet to be fully understood. It is well known that single nucleotide polymorphisms (SNPs), common sources of human genetic variation, may contribute to an individual’s susceptibility to cancer, including NHL [3-5].

Genomic DNA damage caused by exposure to either endogenous or exogenous toxic substances, if not repaired, may lead to tumorigenesis. DNA repair pathways continuously correct damaged DNA to maintain genomic stability and homeostasis [6]. Defects in DNA repair pathways are closely associated with excessive cell death or the malignant transformation of cells, which may play a critical role in the progression of cancer [7]. Nucleotide excision repair (NER) is considered the most versatile DNA repair mechanism and is responsible for removing a wide variety of DNA lesions, including bulky adducts, crosslinks, oxidative DNA damage, alkylating damage, and thymine dimers [8]. This pathway involves an at least four-step reaction (i.e., damage recognition, damaged DNA incision, gapped DNA repair, and DNA ligation) and several key enzymes, including xeroderma pigmentosum complementation group D (XPD) [9]. Polymorphisms in the NER pathway may be implicated in carcinogenesis due to their potential to alter the DNA repair capacity of the host [10].

*XPD*, also known as excision repair cross-complementation group 2 (*ERCC*2), is located at chromosome 19q13.3. It is one of the seven genetic complementation groups that encode for components of the NER pathway, which restores DNA damage caused by ionizing radiation and chemotherapy [10,11]. There are two widely investigated polymorphisms in the coding region of *XPD*: Asp312Asn and Lys751Gln. The Asp312Asn polymorphism (rs1799793 G > A) at position 312 in exon 10 results in an amino acid substitution from aspartic acid (Asp) to asparagine (Asn), whereas the Lys751Gln polymorphism (rs13181 A > C) at position 751 in exon 23 causes an amino acid substitution from lysine (Lys) to glutamine (Gln) [12].

Currently, several studies have investigated the association of the Asp312Asn [13-18] or Lys751Gln [13-19] *XPD* polymorphism with NHL risk, but the conclusions were inconclusive. Therefore, we performed this meta-analysis of all eligible case-control studies to provide an updated and more precise estimation of these associations.

## Methods

### Literature search strategy

A comprehensive literature search was performed to identify publications reporting on the association of the Asp312Asn or Lys751Gln polymorphism in the *XPD* gene with NHL risk. We searched the electronic literature in the PubMed, Embase, and Chinese Biomedical (CBM) databases for all relevant studies using the following key words: “*XPD* or xeroderma pigmentosum group D or *ERCC*2 or excision repair cross-complementation group 2”, “variant or variation or polymorphism”, and “NHL or non-Hodgkin lymphoma or non-Hodgkin’s lymphoma” (last updated March 28, 2014). All available publications were retrieved to evaluate their eligibility. The reference lists of the retrieved articles were also hand searched to find additional relevant publications. Only publications with the full text available were included.

### Selection criteria

The inclusion criteria used in this meta-analysis were as follows: 1) evaluation of the association of the Asp312Asn and/or Lys751Gln polymorphisms of the *XPD* gene with NHL risk; 2) case-control, nested case-control, or cohort study; 3) sufficient data provided to estimate odds ratios (ORs) and 95% confidence intervals (CIs); 4) original genotyping data available from the data source; 5) English or Chinese language; and 6) agreement of the genotyping data of the controls with Hardy-Weinberg equilibrium (HWE).

The following exclusion criteria were adopted: 1) incomplete raw data; 2) repetitive reports (only the most recent report or the report containing the largest sample size was selected if more than one publication on the same study was retrieved); 3) the lack of a case-control design; and 4) departure from HWE without further evidence from other SNPs in the *XPD* gene.

### Data extraction

The following information was collected from each study: the first author’s surname, publication year, country of origin, ethnicity, source of the controls, genotyping methods, subtypes of NHL, total number of cases and controls, and genotype counts for the cases and the controls: the GG, GA, and AA genotypes for Asp312Asn (rs1799793 G > A) and the AA, AC, and CC genotypes for Lys751Gln (rs13181 A > C).

Subgroup analysis was conducted after stratifying by ethnicity (Asians, Caucasians, and mixed ethnicity), source of the controls (hospital-based and population-based), or the NHL subtypes (DLBCL and FL).

### Statistical methods

The strength of the association between the Asp312Asn or Lys751Gln polymorphism and NHL risk was assessed by calculating ORs and corresponding 95% CIs. Briefly, the pooled ORs were estimated for Asp312Asn under the homozygous (AA vs. GG), heterozygous (GA vs. GG), recessive [AA vs. (GA + GG)], and dominant [(GA + AA) vs. GG] models, as well as via allele comparison (A vs. G). Similarly, the pooled ORs for Lys751Gln were calculated under the homozygous (CC vs. AA), heterozygous (AC vs. AA), recessive [CC vs. (AC + AA)], and dominant [(AC + CC) vs. AA] models, as well as via allele comparison (C vs. A). Subgroup analyses were further performed according to ethnicity, source of the controls, and NHL subtype.

The Chi square-based *Q*-test was used to assess between-study heterogeneity. If there was no heterogeneity (*P* > 0.10), the fixed-effect model (the Mantel–Haenszel method) was used [20]. Otherwise, the random-effect model (the DerSimonian and Laird method) was used [21]. The log of the standard error was plotted against the log for each publication to detect potential publication bias. Moreover, we assessed the asymmetry of the funnel plot using Egger’s linear regression test. [22] Sensitivity analyses were conducted to assess the effect of each study on the results of NHL risk by excluding each investigation and recalculating the ORs and the 95% CIs for the remaining studies.

The analyses were performed using STATA software (version 11.0; Stata Corporation, College Station, TX, USA). All *P* values were two-sided, and *P* values less than 0.05 were considered significant.

## Results

### Study characteristics

A total of 11 potentially relevant publications were identified from the PubMed, Embase, and CBM databases. After assessment of the abstracts and the full texts, 2 publications [23,24] were excluded due to the lack of relevance, and an additional 2 publications [25,26] were excluded because they overlapped with other studies [13,14,16]. Ultimately, 7 publications met the inclusion criteria and were included in the final meta-analysis (Table 1). Of them, 6 publications [13-18] examined both Asp312Asn and Lys751Gln and 1 [19] examined only Lys751Gln.

**Table 1:** **Characteristics of the included case-control studies reporting on the association of the Asp312Asn or Lys751Gln polymorphism in the xeroderma pigmentosum complementation group D (** ***XPD*** **) gene with the risk of non-Hodgkin’s lymphoma (NHL)**

**Reference**	**Year**	**Country**	**Ethnicity**	**Source of controls**	**Genotyping method**	**Cases**				**Controls**				**MAF**	**HWE**
Asp312Asn (rs1799793 G > A) polymorphism						GG	GA	AA	All	GG	GA	AA	All		
Hill *et al*. [13]	2006	USA	Mixed	PB	TaqMan	355	306	86	747	307	273	56	636	0.303	0.671
Shen *et al*. [14]	2006	USA	Mixed	PB	TaqMan	199	189	57	445	226	238	70	534	0.354	0.557
Smedby *et al*. [15]	2006	Denmark and Sweden	Caucasian	PB	Mass spectrometry	167	211	50	428	262	255	85	602	0.353	0.075
Shen *et al*. [16]	2007	Australia	Caucasian	PB	TaqMan	272	210	72	554	238	211	52	501	0.314	0.606
Song *et al*. [17]	2008	China	Asian	HB	PCR-RFLP	256	47	4	307	265	35	3	303	0.068	0.142
Worrillow *et al*. [18]	2009	UK	Caucasian	PB	TaqMan	270	265	79	614	316	335	79	730	0.338	0.483
Lys751Gln (rs13181 A > C) polymorphism						AA	AC	CC	All	AA	AC	CC	All		
Hill *et al*. [13]	2006	USA	Mixed	PB	TaqMan	320	333	93	746	278	295	66	639	0.334	0.343
Shen *et al*. [14]	2006	USA	Mixed	PB	TaqMan	203	189	64	456	207	256	67	530	0.368	0.375
Smedby *et al*. [15]	2006	Denmark and Sweden	Caucasian	PB	Mass spectrometry	159	209	56	424	231	254	106	591	0.394	0.015
Shen *et al*. [16]	2007	Australia	Caucasian	PB	TaqMan	239	229	74	542	210	211	57	478	0.340	0.720
Song *et al*. [17]	2008	China	Asian	HB	PCR-RFLP	261	43	5	309	270	32	3	305	0.062	0.075
Worrillow *et al*. [18]	2009	UK	Caucasian	PB	TaqMan	286	329	85	700	294	378	107	779	0.380	0.405
Yang *et al*. [19]	2009	China	Asian	HB	Mass Array	64	7	1	72	304	48	2	354	0.073	0.944

MAF, minor allele frequency; HWE, Hardy–Weinberg equilibrium; USA, the United States of America; UK, the United Kingdom; PB, population-based; HB, hospital-based; PCR-RFLP, polymerase chain reaction-restriction fragment length polymorphism.

Overall, 3,095 NHL cases and 3,306 controls for Asp312Asn and 3,249 NHL cases and 3,676 controls for Lys751Gln were included in the final meta-analysis. Three studies were conducted on Caucasians, 1 on Asians, and 2 on mixed ethnicities for the Asp312Asn polymorphism; 3 were conducted on Caucasians, 2 on Asians, and 2 on mixed ethnicities for the Lys751Gln polymorphism. With respect to the source of the controls, for Asp312Asn, 5 studies were population-based and 1 was hospital-based; for Lys751Gln, 5 studies were population-based and 2 were hospital-based. With respect to the NHL subtype, for Asp312Asn, 4 studies examined DLBCL and 5 examined FL, whereas for Lys751Gln, 5 studies examined DLBCL and 6 examined FL.

### Meta-analysis results of Asp312Asn polymorphism

**Figure 1:**
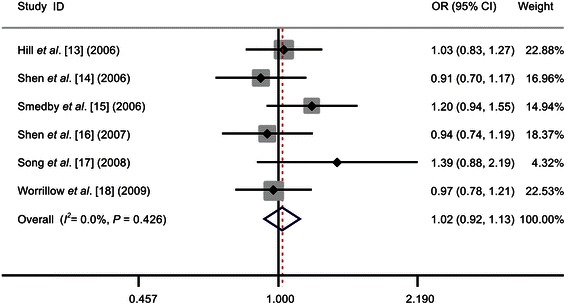
**Forest plots of the effect estimates for the association between the Asp312Asn polymorphism in the xeroderma pigmentosum complementation group D (** ***XPD*** **) gene and the risk of non-Hodgkin’s lymphoma (NHL) under the dominant model.** No significant association was detected between the Asp312Asn polymorphism and NHL risk. For each study, the estimates of the odds ratio (OR) and the 95% confidence interval (CI) are indicated by a box and a horizontal line, respectively. ◇, pooled OR and its 95% CI.

As shown in Table 2 and Figure 1, our meta-analysis did not reveal any significant association between the Asp312Asn polymorphism and NHL risk under any genetic model (homozygous: OR = 1.11, 95% CI = 0.94-1.32; heterozygous: OR = 1.00, 95% CI = 0.89-1.11; recessive: OR = 1.12, 95% CI = 0.95-1.31; dominant: OR = 1.02, 95% CI = 0.92-1.13; and allele comparison: OR = 1.04, 95% CI = 0.96-1.12).

**Table 2:** M**eta-analysis of the association between each** ***XPD*** **gene polymorphism and NHL risk**

**Variable**	**No. of studies**	**Sample size (cases/controls)**	**Homozygous**		**Heterozygous**		**Recessive**		**Dominant**		**Allele comparison**	
			**OR (95% CI)**	***P*** ^**het**^	**OR (95% CI)**	***P*** ^**het**^	**OR (95% CI)**	***P*** ^**het**^	**OR (95% CI)**	***P*** ^**het**^	**OR (95% CI)**	***P*** ^**het**^
Asp312Asn polymorphism			AA vs. GG		GA vs. GG		AA vs. (GA + GG)		(GA + AA) vs. GG		A vs. G	
All	6	3,095/3,306	1.11 (0.94-1.32)	0.718	1.00 (0.89-1.11)	0.935	1.12 (0.95-1.31)	0.368	1.02 (0.92-1.13)	0.426	1.04 (0.96-1.12)	0.717
Ethnicity												
Caucasian	3	1,596/1,833	1.10 (0.88-1.37)	0.580	1.00 (0.87-1.16)	0.073	1.09 (0.89-1.34)	0.156	1.02 (0.89-1.17)	0.315	1.03 (0.93-1.14)	0.986
Asian	1	307/303	1.38 (0.31-6.23)	NA	1.39 (0.87-2.22)	NA	1.32 (0.29-5.95)	NA	1.39 (0.88-2.19)	NA	1.36 (0.89-2.07)	NA
Mixed	2	1,192/1,170	1.12 (0.86-1.46)	0.192	0.94 (0.79-1.12)	0.687	1.16 (0.90-1.50)	0.217	0.98 (0.83-1.15)	0.450	1.02 (0.90-1.15)	0.267
Source of the controls												
PB	5	2,788/3,003	1.11 (0.94-1.32)	0.591	0.98 (0.88-1.09)	0.223	1.12 (0.95-1.31)	0.252	1.00 (0.91-1.12)	0.549	1.03 (0.95-1.11)	0.864
HB	1	307/303	1.38 (0.31-6.23)	NA	1.39 (0.87-1.22)	NA	1.32 (0.29-5.95)	NA	1.39 (0.88-2.19)	NA	1.36 (0.89-2.07)	NA
NHL subtype												
DLBCL	4	726/2,170	1.00 (0.73-1.37)	0.829	0.98 (0.80-1.18)	0.667	1.01 (0.75-1.37)	0.669	1.02 (0.86-1.22)	0.463	0.99 (0.86-1.14)	0.998
FL	5	1,088/2,772	1.12 (0.88-1.42)	0.595	1.00 (0.85-1.17)	0.059	1.10 (0.88-1.38)	0.194	1.03 (0.89-1.20)	0.297	1.04 (0.93-1.16)	0.732
Lys751Gln polymorphism			CC vs. AA		AC vs. AA		C/C vs. (AC + AA)		(AC + CC) vs. AA		C vs. A	
All	7	3,249/3,676	0.97 (0.83-1.15)	0.428	0.96 (0.86-1.06)	0.184	1.00 (0.86-1.16)	0.200	0.96 (0.87-1.06)	0.335	0.98 (0.91-1.05)	0.405
Ethnicity												
Caucasian	3	1,666/1,848	0.88 (0.72-1.09)	0.305	0.99 (0.86-1.14)	0.255	0.88 (0.72-1.07)	0.137	0.96 (0.84-1.10)	0.482	0.95 (0.86-1.05)	0.522
Asian	2	381/659	1.85 (0.53-6.47)	0.823	1.15 (0.76-1.73)	0.159	1.81 (0.52-6.33)	0.777	1.20 (0.81-1.78)	0.184	1.23 (0.85-1.77)	0.232
Mixed	2	1,202/1,169	1.11 (0.85-1.44)	0.397	0.88 (0.74-1.05)	0.140	1.19 (0.93-1.52)	0.718	0.93 (0.79-1.09)	0.140	1.00 (0.89-1.13)	0.227
Source of the controls												
PB	5	2,868/3,017	0.96 (0.82-1.13)	0.307	0.94 (0.84-1.05)	0.205	0.99 (0.85-1.15)	0.111	0.95 (0.85-1.05)	0.438	0.97 (0.90-1.04)	0.530
HB	2	381/659	1.85 (0.53-6.47)	0.823	1.15 (0.76-1.73)	0.159	1.81 (0.52-6.33)	0.777	1.20 (0.81-1.78)	0.184	1.23 (0.85-1.77)	0.232
NHL subtype												
DLBCL	5	946/2,731	0.96 (0.74-1.24)	0.003	0.90 (0.76-1.07)	0.213	1.01 (0.79-1.29)	0.005	0.95 (0.81-1.11)	0.042	0.96 (0.85-1.08)	0.005
FL	6	1,227/3,322	0.87 (0.69-1.08)	0.522	0.95 (0.82-1.11)	0.234	0.88 (0.72-1.09)	0.227	0.95 (0.82-1.09)	0.448	0.93 (0.84-1.03)	0.681

OR, odds ratio; CI, confidence interval; *P* ^het^, *P* value for heterogeneity; NA, not applicable; DLBCL, diffuse large B-cell lymphoma; FL, follicular lymphoma. Other abbreviations as in Table 1.

Upon stratifying the data by ethnicity, source of the controls, and NHL subtype, no association between the Asp312Asn polymorphism and NHL risk was detected.

### Meta-analysis results of Lys751Gln polymorphism

**Figure 2:**
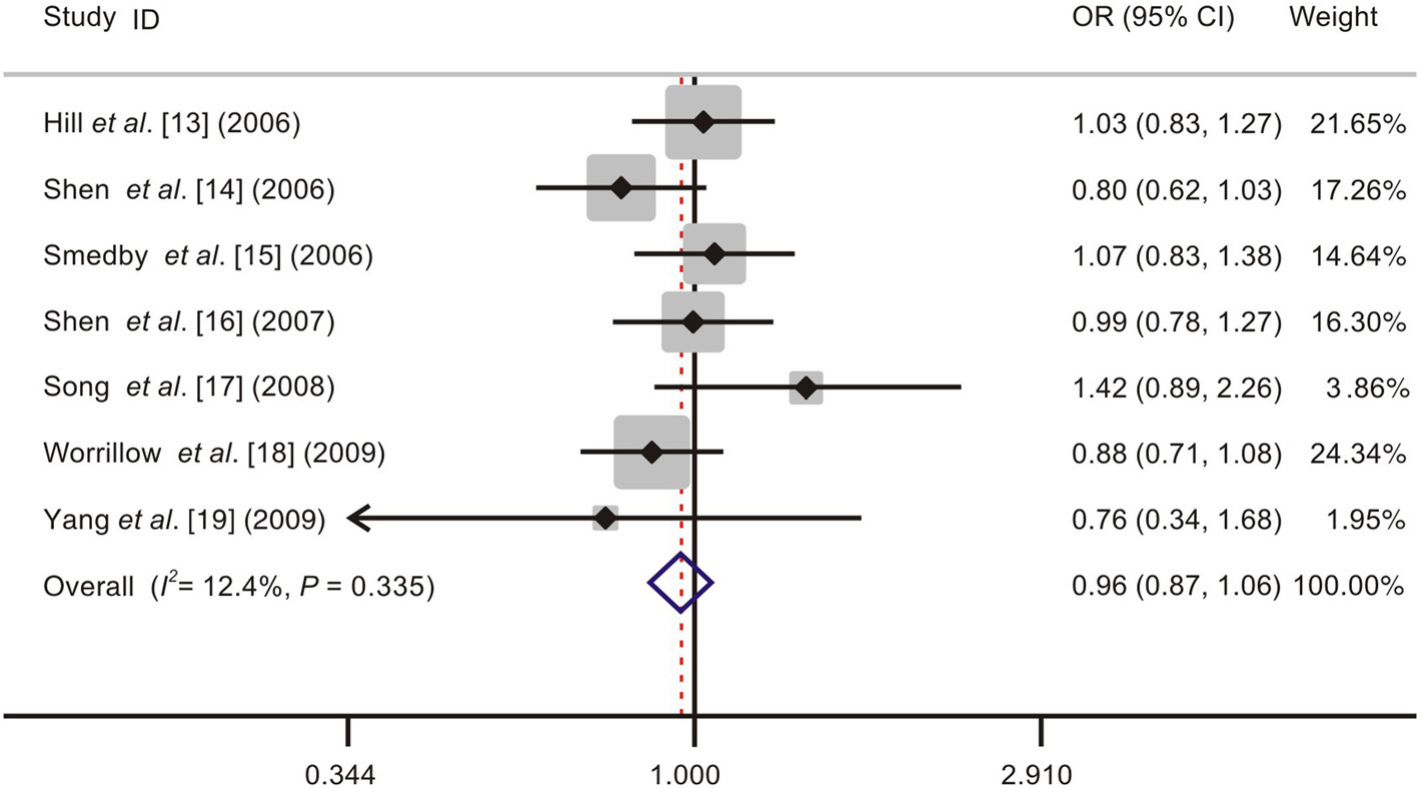
**Forest plots of the effect estimates for the association between the Lys751Gln polymorphism in the** ***XPD*** **gene and NHL risk under the dominant model.** No significant association was detected between the Lys751Gln polymorphism and NHL risk. For each study, the estimates of the OR and the 95% CI are indicated by a box and a horizontal line, respectively. ◇, pooled OR and its 95% CI.

Similar to the Asp312Asn polymorphism, the Lys751Gln polymorphism did not display any significant association with NHL risk (homozygous: OR = 0.97, 95% CI = 0.83-1.15; heterozygous: OR = 0.96, 95% CI = 0.86-1.06; recessive: OR = 1.00, 95% CI = 0.86-1.16; dominant: OR = 0.96, 95% CI = 0.87-1.06; and allele comparison: OR = 0.98, 95% CI = 0.91-1.05) (Table 2 and Figure 2).

Moreover, based on the subgroup analyses, ethnicity, source of the controls, and NHL subtype had no effect on the association between the Lys751Gln polymorphism and NHL risk.

### Heterogeneity and sensitivity analyses

No heterogeneity was observed among all studies for the Asp312Asn polymorphism (homozygous: *P* = 0.718; heterozygous: *P* = 0.935; recessive: *P* = 0.368; dominant: *P* = 0.426; and allele comparison: *P* = 0.717) or Lys751Gln polymorphism of the *XPD* gene (homozygous: *P* = 0.428; heterozygous: *P* = 0.184; recessive: *P* = 0.200; dominant: *P* = 0.335; and allele comparison: *P* = 0.405). Therefore, the fixed-effect model was chosen for the analyses using all genetics models.

The leave-one-out sensitivity analysis indicated that no single study clearly altered the pooled ORs.

### Publication bias

The shapes of the funnel plots for the Asp312Asn (homozygous: *P* = 0.893; heterozygous: *P* = 0.249; recessive: *P* = 0.955; dominant: *P* = 0.209; and allele comparison: *P* = 0.195) and Lys751Gln polymorphisms (homozygous: *P* = 0.298; heterozygous: *P* = 0.825; recessive: *P* = 0.393; dominant: *P* = 0.689; and allele comparison: *P* = 0.523) of the *XPD* gene were symmetrical, indicating that there was no evidence of publication bias among the studies included in the meta-analysis.

## Discussion

The associations of the Asp312Asn and Lys751Gln polymorphisms of the *XPD* gene with NHL risk have been investigated by different research groups, but the conclusions were contradictory. The most possible reason for the discrepancies between studies is the small sample size of each study, which limits the statistical power to detect the potential effects of polymorphisms. We performed the present meta-analysis via a systematic literature search to combine the results of all available studies, which may be useful for evaluating the genetic factors that contribute to NHL. In this meta-analysis, which included 3,095 cases and 3,306 controls for Asp312Asn and 3,249 cases and 3,676 controls for Lys751Gln, we found that neither the Asp312Asn nor the Lys751Gln polymorphism was significantly associated with NHL risk. The subgroup analysis also failed to reveal any association between the examined polymorphisms and NHL risk according to ethnicity, source of the controls, or NHL subtype.

The tumorigenesis of NHL is a complex multi-step process that leads to clonal, uncontrolled, malignant lymphocyte proliferation. Well-established pathogenic factors of NHL include environmental and microbial factors, as well as immune disorder, which may result in malignant transformation. For instance, exposure to environmental carcinogens can result in various types of DNA damage that subsequently induce the development of NHL [27]. In addition, an increasing number of studies indicate that genetic aberration may play a critical role in the progression of NHL [28]. Certain NER pathway polymorphisms, such as Ala499Val and Lys939Gln in the *XPC* gene, have been shown to associate with overall cancer risk [29]. XPD, a helicase that unwinds DNA in the 5' to 3' direction, plays a critical role in opening the DNA helix during the process of NER [30]. The XPD protein directly interacts with the basal transcription factor IIH (TFIIH) complex, which greatly enhances the helicase activity of XPD by 10 folds. Activated XPD then unwinds the DNA helix to facilitate the excision of the damaged DNA fragment during transcription-coupled repair [31]. These amino acid substitutions in the conserved region of XPD may influence its function as a helicase, which might ultimately impede DNA repair capacity and increase cancer risk. Mutation of the *XPD* gene may weaken the interaction between the TFIIH complex and XPD, abolishing the stimulation of the helicase activity of XPD, thereby reducing DNA repair and transcription capacity and producing abnormal responses to apoptotic signals [32].

*XPD* gene polymorphisms have been shown to associate with the risk of a wide range of cancers, including gastric cancer [33], breast cancer [34], bladder cancer [35], and so on. In previous studies, the Lys751Gln polymorphism has been shown to decrease DNA repair capacity, and 751Gln alleles were associated with decreased NER capacity compared with wild-type alleles [36]. In this meta-analysis, our results did not support a genetic association of either the Asp312Asn or the Lys751Gln polymorphism with NHL risk. This lack of an association may be due to the relatively small sample size of the present meta-analysis. Alternatively, these results suggest that the influence of the examined polymorphisms on cancer is tissue-specific.

There are several limitations in our meta-analysis that remain to be addressed. To date, there are only 6 studies for Asp312Asn and 7 for Lys751Gln that have investigated the association between these polymorphisms and NHL risk. Moreover, the sample sizes of most of these studies are relatively small. As a result, this meta-analysis may have limited statistical power to detect a potential association. In addition, different genotyping methods with differing accuracy were used in previous investigations, which might lead to a bias to some extent. Additionally, because other detailed data, such as age, sex, smoking habits, and alcohol consumption, were not available, our conclusions were solely based on unadjusted estimated ORs.

## Conclusion

In conclusion, this meta-analysis suggests that there is no significant association of either the Asp312Asn or the Lys751Gln polymorphism of the *XPD* gene with NHL risk. Large, well-designed, prospective studies are required to verify our findings.
